# Phenotypic Bacterial Isolates, Antimicrobial Susceptibility pattern and Associated factors among Septicemia Suspected Patients at a hospital, in Northwest Ethiopia: Prospective cross-sectional study

**DOI:** 10.1186/s12941-023-00599-7

**Published:** 2023-06-22

**Authors:** Habtamu Belew, Workineh Tamir, Tebelay Dilnessa, Abeba Mengist

**Affiliations:** 1 Department of Medical Laboratory Science, College of health sciences, Debre Markos University, 269, Debre Markos, Ethiopia; 2 Department of Medical Laboratory sciences, College of health sciences, Injibara University, 40, Injibara, Ethiopia

**Keywords:** Bacterial isolates, Septicemia, Antimicrobials susceptibility, Ethiopia

## Abstract

**Background:**

Septicemia is potentially fatal infection caused by pathogenic bacteria infiltrating the bloodstream, resulting in morbidity and mortality among Ethiopian hospital patients. Multidrug resistance is a therapeutic challenge in this patient population. There is an insufficiency data among hospitals in Ethiopia. Hence, this study aimed to assess the phenotypic bacterial isolates, antimicrobial susceptibility pattern, and associated factors among septicemia suspected patients.

**Methods:**

Prospective cross-sectional study was conducted among 214 septicemia suspected patients from February to June 2021 at Debre Markos Comprehensive Specialized hospital in northwest, Ethiopia. Blood samples were collected aseptically and processed to identify bacterial isolates by using different standard microbiological procedures. Antimicrobial susceptibility pattern was performed using the modified Kirby Bauer disc diffusion on Mueller Hinton agar. Epi-data V4.2 was used to enter data and SPSS V25 for analysis. The variables were assessed using a bivariate logistic regression model with a 95% confidence interval, and declared statistically significant; P-value was < 0.05.

**Results:**

The overall bacterial isolates was found 45/214 (21%) in this study. Gram-negative and positive bacteria were 25/45(55.6%), 20/45(44.4%) respectively. The most common bacterial isolates were *Staphylococcus aureus12/45* (26.7%), *Klebsiella pneumoniae* 8/45(17.8%), *Escherichia coli 6/45* (13.3%). Gram-negative bacteria showed susceptibility to amikacin (88%), meropenem, imipenem (76%) but, (92%) resistance to ampicillin, (85.7%) amoxicillin-clavulanic acid. *S.aureus* (91.7%) was resistance to Penicillin, (58.3%) cefoxitin and (75%) susceptible to ciprofloxacillin. *S.pyogenes* and *S.agalactia* were (100%) susceptible to Vancomacin. Multidrug resistance was found in 27/45(60%) of the bacterial isolates. The main predictors related to patients suspected of septicemia were prolonged hospitalization (AOR = 2.29, 95% CI: 1.18, 7.22), fever (AOR = 0.39, 95%CI: 0.18, 0.85) and length of hospital stay (AOR = 0.13, 95%CI: 0.02, 0.82).

**Conclusions:**

Incidence of bacterial isolates among septicemia suspected patients were high. The majority of the bacterial isolates were multidrug-resistant. To prevent antimicrobial resistance, specific antibiotic utilization strategy should be applied.

## Background

Septicemia is a life-threatening bloodstream infection that is a major public health concern around the world if not treated with appropriate antimicrobials [1]. Septicemia is when the bacteria enter the bloodstream, and cause blood poisoning which triggers sepsis and comparable with bacteremia with sepsis, and systemic inflammatory response syndrome (SIRS) [2, 3]. It causes illness and deaths in all group of population [4], especially in immunocompromised patients, an intensive care unit (ICU), elders, and children [5], cancer patients [6, 7], and patients living with human immunodeficiency virus (HIV) [8]. The incidence rate of septicemia is increased due to previous hospitalization, length of hospital stay, unexplained fever (> 37^o^c), age groups, serious injury, chronic antibacterial therapy, comorbidities, invasive medical procedures, respiratory disease, urinary tract disease, gastrointestinal disease, HIV/AIDS, hematological disorders are the factors that increase the severity of septicemia [4, 6, 9, 10]. Different Gram-negative and Gram-positive bacteria are common, and vary depending on geographical location and time [11–14].

According to recent scientific publications, the global burden of bacterial infection of the blood was 48.9 million new cases of septicemia in 2017, with 11.0 million septicemia-related deaths, accounting for 20% of all deaths worldwide [15]. The World Health Organization (WHO) estimates that 41% of all global sepsis cases in 2020 were children under the age of five, with a death rate of 42% among intensive care patients with septicemia [16, 17]. One in two cases of sepsis in intensive care units (ICUs) results from healthcare-associated (HCA) infections. Mortality in HCA septicemia from hospitalized adult patients ranges from 20 to 30%, and in pediatric ICUs from 20 to 50% of deaths worldwide among 200,000 cases reported [16–20]. On the other hand, 40% of infants diagnosed with septicemia were died, with the majority of deaths occurring in developing countries, making it difficult to control worldwide [21, 22]. More than 19 million septicemia cases and 5 million septicemia-related deaths are estimated to occur each year in low and middle-income countries including Ethiopia [15, 23].

Antimicrobial management is initiated empirically in almost all septicemia-related cases prior to the results of blood culture [22]. As a result, patients who are improperly treated may have a 100% mortality rate [24] and the emergence of antimicrobial resistance in all types of pathogenic bacteria has worsened in resource-constrained countries [25, 26]. Furthermore, self-prescription, misuse, and over-prescription of antimicrobials in the absence of true etiological agent identification pose a significant challenge to antimicrobial resistance control in Ethiopia. As a result, there is little information available about commonly prescribed antibiotics and their susceptibility profiles, as well as the epidemiology of bacterial isolates from suspected septicemia patients. Thus, this study aimed to assess the phenotypic bacterial isolates, antimicrobial susceptibility pattern, and associated factors among septicemia suspected patients at a hospital, in Northwest Ethiopia.

## Methods

### Study design, period and settings

A hospital-based prospective cross-sectional study was conducted in septicemia suspect patients referred and admitted to Debre Markos Comprehensive Specialized Hospital (DMCSH) from February to June 2021. The hospital is located in Amhara National Regional state’s East Gojjam zone, at Debre Markos town. DMCSH is a 218-bed hospital and the largest public comprehensive hospital in the zone, receiving patients directly from the community as well as referrals from district primary hospitals and health centers. DMCSH provides health services to over 5 million people in catchment area, and neighboring border neighborhoods. It has various wards of ICU, medical, pediatric, surgery, emergency, gynecology, and medical laboratory services.

### Inclusion criteria

During the study period, the study populations were included when all patients have over the age of one month who was suspected of having septicemia and given consent and assent.

### Exclusion criteria

Patients who were seriously ill and did not provide a blood sample during the data collection period, contaminated blood samples, were excluded from the study.

#### Sample size determination and sampling

The sample size was calculated with the formula (n= (Z_2_)^2^ P (1- P) /d^2^). By reviewing similar previous studies, the maximum sample size was obtained from a study conducted in southern Ethiopia, with a prevalence/proportion of septicemia was 15.8% (0.158) [1]. As a result, n= (Z_2_)^2^ P (1- P) /d^2^ with a margin of error (d = 0.05) and a 95% confidence interval. p = 0.158, d = 0.05, Z = 0.05 = Z/2 = 0.025 = 1.96. As a result, n= (1.96)^2^* 0.158(1-0.158)/ (0.05)^2^ =204. Then a 5% non-respondent rate was added, the total sample size was 214. A convenience sampling technique was used, when attending physicians suspected septicemia on each ward.

#### Operational definition

##### Multidrug resistance:

Multidrug resistance is defined in this study as simultaneous resistance to three or more antimicrobial agents from different antimicrobial groups [27].

**Septicemia:** when the patients classified as having septicemia the physician suspected two or more symptoms of SIRS, temperature > 38°c or < 36°c heart rate > 90/min, respiratory rate > 20/min and white blood cell count > 12,000/mm^3^ or < 4000/mm^3^ to the infection [2].

**Fever:** The patients have greater than > 37°c during the study period (yes or no question).

### Data collection, specimen processing and culturing for identification of bacterial isolates

To collect socio-demographic, laboratory, and clinical data, semi-structured questionnaires were prepared from different previous literatures [4, 9, 10, 12, 13, 28]. **Face-to-face interviews with trained nurses and laboratory personnel were used**. The blood samples were collected by laboratory technicians and experienced nurses who had undergone training. The vein puncture site was properly disinfected with 70% alcohol and 2% iodine tincture. Within a 30-minute interval, two bottles of blood (5 mL of blood for patients over the age of five and 1–3 mL of blood for patients under the age of five) were collected aseptically from both hands for each patient [29]. After obtaining the blood, it was inoculated at bedside into Tryptone Soy Broth (TSB) (Oxoid, Hampshire, UK) maintaining a minimum of blood to broth ratio 1:10. The TSBs were prepared according to the blood specimen collected; 10ml, 20ml, 30ml, and 45ml. Medical microbiology specialists were performed blood culture processing, biochemical testing, and antimicrobial susceptibility pattern of the isolate. The blood bottles were incubated at 37°c for 7 days and were inspected daily for the presence of visible microbial growth. Sub-cultures were made after 24 h, 48 h, and 72 h on sheep blood agar plate (SBAP) (Oxoid, Hampshire, UK), chocolate agar plate (CAP) (Oxoid, Hampshire, UK), MacConkey agar plate (MACA) (Oxoid, Hampshire, UK), and Mannitol salt agar (MSA) (Oxoid, Hampshire, UK) (Oxoid, Hampshire, UK). The BAP, MACA, and MSA plates were incubated in an aerobic atmosphere at 37^0^c and examined after 24–48 h, whereas the CAP plates were incubated in a candle jar at 5–10% CO_2_ at the same temperature. Finally, on the 7th day, blood bottles that did not show bacterial growth were reported as having no bacterial growth when sub-cultures were made on SBAP, MACA and CAP and gram stains were negative. Bacterial growths on sub-cultured plates were recognized by their distinct appearances. For bacterial isolates, Gram stain and specific biochemical reaction panels were used for identification. Gram-negative bacteria were identified using biochemical tests; the indole test (Hampshire, UK), triple sugar iron (TSI) test (Hampshire, UK), citrate utilization (Hampshire, UK), motility test (Hampshire, UK), lysine decarboxylase (Hampshire, UK), urease test, hydrogen sulphide (H2S) production, Methyl red, voges proskarer and oxidase test. Gram-positive bacteria were tested using catalase, coagulase, the CAMP test, and the Bacitracin test.

### Antimicrobial susceptibility testing

All blood culture isolates were tested for antimicrobial susceptibility using modified Kirby Bauer disc diffusion methods on Mueller-Hinton agar (MHA) plates (Oxoid, Hampshire, UK) using clinical and Laboratory standards Institute (CLSI ) 2021 guideline [30]. Fresh 2 to 5 pure colonies from BAP or CAP were selected and transferred to 5 ml of sterile nutrient broth (Oxoid, Hampshire, UK.) and mixed to make the suspension homogeneous before being transferred into sterile normal saline with a sterile pipette. Finally, turbidity was visually adjusted with sterile normal saline to match a 0.5 McFarland standard. Using a sterile swab, the suspension was then inoculated uniformly over the entire surface of an MHA plate. For 3–15 min, the inoculated plates were left at room temperature to dry. The antibiotic discs were placed on MHA using sterile forceps at a distance of 24 mm between each disk and 15 mm from the border. The antimicrobial discs were used in the concentrations indicated in brackets, were chosen based on availability, and were frequently prescribed for the treatment of bacterial infections in hospitals throughout Ethiopia. Antibiotics used for this study were: Ampicillin (AMP)(10 µg), ceftazidime(CAZ)(30 µg), ceftriaxone (CTR) (30 µg), cefepime (CFP) (30 µg) chloramphenicol (CHL) (30 µg), ciprofloxacin (CPR) (5 µg), gentamicin(GEN) (10 µg), amikacin (AMK) (30 µg), piperacillin(PIP) (10 µg), meropenem(MER)(10 µg),imipenem (IMP)(10 µg) and trimethoprinsulfamethoxazole(TS)(1.25/23.75 µg) ,amoxicillin- clavulanic acid(AMC) (20 µg),cefoxitin (CXT) (30 µg),penicillin (P) (10 IU), and vancomacin (VAN) (30 µg). Cefoxitin disk diffusion tests were used to identify methicillin resistance *Staphylococci*. The results were interpreted by measuring the zone of inhibition as sensitive, intermediate, and resistant according to the standardized CLSI 2021 guidelines [30].

### Quality control

Questionnaires had been pre-tested on 5% questionnaires from fenote Selam General Hospital to ensure consistency. The questionnaires were written in English, translated into Amharic, and then back translated into English. A half-day training session was provided for laboratory personnel and nurses. Standard operating procedures (SOPs) were strictly followed throughout the laboratory analysis in this study to ensure the quality of the process from pre-analytical to post-analytical. To ensure the sterility of the media, 5% of all prepared culture media were incubated overnight at 37 °C without inoculation. A performance check was made on standard reference strains to the culture media of BAP, CAP, MACA, and MSA whenever a new batch of media was prepared. American Type Culture Collection (ATCC) standard reference strains (*E. coli* ATCC 25,922, *S. aureus* (ATCC 25,923, and *P.aeruginosa* ATCC 27,853) were used. The principal investigator daily checked the completeness of data and the laboratory work procedures as a whole.

### Data analysis and interpretation

The Epi-Data V.4.2 computer program was used to enter and clean the data before exporting it to the SPSS V.25 software package for analysis. Cross-tabulation of each variable was used to ensure the data’s completeness and consistency. Descriptive analysis was used like tables, graphs. A binary logistic regression model was used to demonstrate any relationship between independent and outcome variables. In the bivariate analysis, a variable with a p-value of ≤ 0.25 was entered into the multivariate analysis to identify variables that were independently associated with the outcome variable. The presence and strength of association between independent and outcome variables were calculated using an adjusted odds ratio with a 95% confidence interval (CI). The Hosmer–Lemeshow test was used to assess the model’s fitness test [31]. A p-value of < 0.05 was regarded as an indicator of statistically significant results.

## Results

### Socio-demographic and clinical characteristics

This study was included 214 septicemia suspected patients. Of these, 109 (50.9%) were males. The median age of the participants with their interquartile range was 14 ± 9.3 years, (64%) had fever, and 43 (20.1%) had previous medical procedures (Table 1).

**Table 1:** Socio-demographic characteristics of septicemia suspected patients at a hospital, in Northwest Ethiopia, 2021

Characteristics(n = 214)	Frequency	Percentage
Age		
1mo-<12mo	48	22.4
1–5 years	51	23.8
6–10 years	41	19.2
11–15 years	16	7.5
> 15 years	58	27.1
Sex		
Male	109	50.9
Female	105	49.1
Residence		
Rural	130	60.7
Urban	84	39.3
Patient ward		
Surgery	13	6.1
Emergency	12	5.6
Pediatric	154	72
Medical	21	9.8
ICU	14	6.5
Fever		
Yes	137	64
No	77	36
Temperature		
_<37_^0C^	74	34.6
_>37_^0C^	140	65.4
Previous medical procedure		
Yes	43	20.1
No	171	79.9
Previous hospital admission		
Yes	56	26.2
No	158	73.8
Co morbidity		
Yes	87	40.7
No	127	59.3
Type of chronic disease	18	8.4
HIV/ADIS	20	9.3
Diabetes mellitus	12	5.6
Chronic liver disease	10	4.7
Chronic kidney disease	30	14.5
Chronic heart disease	123	57.5
Wound case		
Yes	36	16.8
No	178	83.2
Burn Case		
Yes	11	5.1
No	203	94.9
Length of hospital stay		
< 5 days	23	10.7
> 5days	191	89.3

### Prevalence of bacterial isolates

**Fig. 1:**
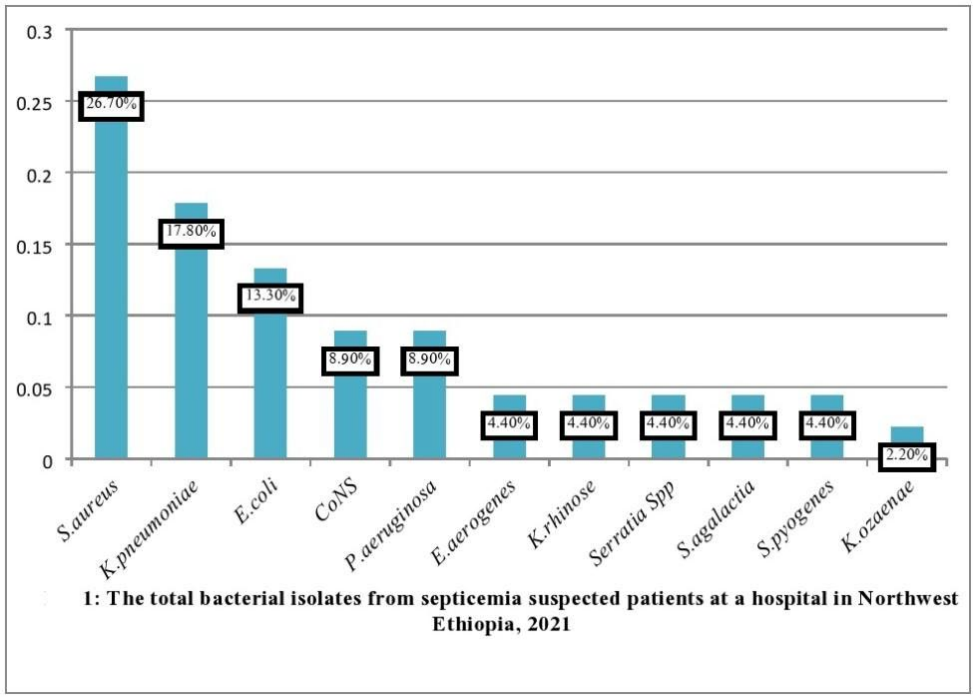
The total bacterial isolates from septicemia suspected patients at a hospital in Northwest Ethiopia, 2021

The overall bacterial isolates from this study was 45/214 (21%, 95% CI; 14.6–25.2%, p = 0.028) for blood culture to different bacterial species. Among the bacterial isolates, Gram-negative bacteria dominated the bacterial isolate, accounting for 25/45(55.6%, 95% CI; 47.2–53.5%) and Gram-positive bacteria accounting for 20/45 (44.4%, 95% CI; 35.1–52.7%). From the Gram-negative bacteria isolated *K.pneumoniae* 8/25(32%), *E.coli* 6/25(24%), *P.aeruginosa* 4/25(16%), *Serratia spp*. 2(8%), *E.aerogenes* 2(8%), *K.rhinose* 2(8%) and *K.ozaenae* 1(4%) were the main bacterial isolates in the blood cultures. Twenty (44.4%) of Gram-positive bacterial isolates were found in this study. *S.aureus* 12/20 (60%), Coagulase-negative *staphylococcus* (CONS) 4/20 (20%), *S.pyogenes* 2/20(10%), and *S.agalactia* 2/20 (10%) were isolated bacteria in septicemia suspected patients. From the total bacterial isolate in the present study *S.aureus* 12/45(26.7%), *K.pneumoniae* 8/45 (17.8%), *E.coli* 6/45 (13.3%), CoNS, and *P.aeroginosa* 4/45 (8.9%) were the main bacterial isolates. All bloodstream bacterial infections isolated during the study period were monomicrobial (Fig. 1).

### Factors associated with blood culture positivity and septicemia

In the bivariate analysis, socio-demographic and clinical variables were revealed to be associated with septicemia in the bivariate. Patients with a temperature greater than 37^0^c and a previous medical procedure had a risk factor, but it was not statistically significant at the 95% CI. In this study, those who had a history of antibiotic use were 2.21 times more likely to have a positive blood culture than those who had never used antibiotics before, and this was statistically significant at (95% CI;1.11–4.39, p = 0.024). The candidate variables for multivariate analysis were age, ward type, fever, previous hospital admission, admission date in the hospital, previous medical procedure, antibiotic usage, and temperature. A multiple logistic regression method using the backward variable selection method revealed that only three variables were associated with blood culture positivity. According to the multiple logistic regression analysis, patients who had previously been admitted to the hospital were 2.9 times more likely to develop septicemia than patients who had never been admitted to the hospital. In this study, patients with fever (temperature > 37^o^C) were 61% less likely to develop septicemia than their counterparts (Table 2).

**Table 2:** Bivariate and multivariate analysis for the socio-demographic and clinical variables associated with septicemia suspected patients at a hospital, in Northwest Ethiopia 2021

Variables	Positive (%) n = 45	Negative (%) n = 169	COR(95%CI)	P-Value	AOR(95%CI)	P-value
Sex						
Male	23(23.8)	89(76.2)	1			
Female	22(19.8)	80(80.2)	0.90(0.46,1.74)	0.753		
Age						
1month-11month	16(33.3)	32(66.7)	0.47(.19,1.14)			
1–5	10(19.6)	41(80.4)	0.96(.37,2.49)	0.25*		
6–10	7(17.1)	34(82.9)	1.14(.40,3.23)			
11–15	2(12.5)	14(87.5)	1.6(.32,8.28)			
> 15	10(19.6)	48(80.4)	1			
Residence						
Rural	14(18.9)	60(81.1)	0.82(.41,1.66)	0.582		
Urban	31(22.1)	109(77.9)	1			
Patient Ward						
Surgery	4(14.3)	24(85.7)	1			
Emergency	5(15.6)	27(84.4)	0.4(.0.04,3.93)	0.432		
Pediatrics	33(29.7)	78(70.3)	0.36(0.04,3.37)	0.371		
Medical	2(7.4)	25(92.6)	0.16(0.02,1.42)	0.079*		
ICU	1(6.3)	15(93.7)	0.83(0.07,9.99)	0.886		
Co morbidity						
Yes	25(21.6)	91(79.6)	1.07(0.55,2.07)	0.84		
No	20(20.4)	78(78.4)	1			
Fever						
Yes	33(23.6)	107(76.4)	1.6(0.3,1.30)	0.026*	0.39(0.18,0.85)	0.012
No	12(16.2)	62(83.8)	1		1	
Hospital admission						
Yes	39(24.7)	119(75.3)	2.73(1.09,6.86)	0.032*	2.9(1.18,7.22)	0.020
No	6(10.7)	50(89.3)	1		1	
Length of hospital stay						
< 5days	11(57.9)	8(42.1)	1		1	0.03
≥ 5days	34(17.8)	157(82.2)	6.35(1.34,42.7)	0.0001*	0.13(0.02,0.82)	
Previous Medical procedure						
Yes	38(23.9)	121(76.1)	2.15(0.90–5.15)	0.085*		
No	7(12.7)	48(87.3)	1			
Antibiotic usage						
Yes	19(13.0)	42(61.8)	2.21(1.11,4.39)	0.024*		
No	26(38.2)	127(87)	1			
Temperature						
< 370c	35(8.8)	108(91.2)	1			
> 370c	10(35.4)	60(64.6)	1.9(.89,4.77)	0.095*		

**Key**: COR: Crude odds ratio, CI: Confidence interval, AOR: Adjusted odds ratio * indicates to the variable entered into multiple logistic regression

### Antimicrobial susceptibility patterns of bacterial isolates

#### The susceptibility of Gram-negatives

Gram-negative bacteria (n = 25) were isolated from blood cultures of patients suspected of having septicemia and tested for sensitivity to 12 antimicrobial discs (Table 3). In this study, the range of sensitivity and resistance of *K. pneumoniae* was 0–87.5% and 0–100%, respectively. *K. pneumoniae* was (100%) resistant to ampicillin and amoxicillin-clavulanic acid, (75%) for Ceftazidime and chloramphenicol, but 7/8 (87.5%) susceptible to amikacin. In the current study, *E. coli* was completely sensitive to amikacin and 83.3% sensitive to meropenem and imipenem. *E.coli* showed high resistance to gentamycin 5/6 (83.3%), ampicillin 5/6 (83.3%), and amoxicillin-clavulanic acid 5/6 (83.3%). *P.aeruginosa* was 100% sensitive to imipenem, 75% sensitive to ciprofloxacillin, amikacin, and meropenem, 75% resistant to gentamycin and piperacillin (Table 3).

**Table 3:** Antimicrobial susceptibility pattern of Gram-negative bacteria isolated from blood culture for patients suspected of septicemia at a hospital, in Northwest Ethiopia, 2021

GNB isolated from blood culture		Antimicrobial disks											
		MER	IPM	AMK	CPR	CTR	GM	C	TS	CAZ	AMP	AMC	PIP
K.pneumoniae (n = 8)	S	5(62.5)	5(62.5)	7(87.5)	4(50)	1(12.5)	3(37.5	2(25)	3(37.5)	2(25)	0	0	
	I	0	1(12.5)	1(12.5)	0	0	0	0	1(12.5)	0	0	0	
	R	3(37.5)	2(25)	0(0.00)	4(50)	7(87.5)	5(62.5	6(75)	4(50)	6(75)	8(100)	8(100)	
E.coli (n = 6)	S	5(83.3)	5(83.3)	6(100)	3(50.0)	3(50.0)	1(16.7	2(33.3)	2(33.30	3(50.0)	1(16.7	1(16.7	
	I	0	0	0	0	0	0	0	0	1(16.7	0	0	
	R	1(16.7)	1(16.7)	0(0.0)	3(50.0)	3(50.0	5(83.3	4(66.7	4(66.7	2(33.3)	5(83.3	5(83.3)	
P.aeruginosa (n = 4)	S	3(75)	4(100)	3(75)	3(75)	Nr	1(25)	Nr	Nr	2(50.0)	1(25)	Nr	1(25)
	I	0	0	0	0	Nr	0	Nr	Nr	0	0	Nr	0
	R	1(25)	0(0.0)	1(25)	1(25)	Nr	3(75)	Nr	Nr	2(50.0)	3(75)	Nr	3(75)
K.rhinose (n = 2)	S	1(50.0)	0(0.0)	1(50)	1(50)	0	0	1(50)	2(100)	0(0.0)	0(0.0)	1(50)	
	I	0	0	0	0	0	0	0	0	0	0	0	
	R	1(50.0)	2(100)	1(50.0)	1(50)	1(50)	2(100	1(50)	0	2(100	2(100	1(50)	
E.aerogenes (n = 2)	S	2(100)	2(100)	2(100)	1(50.0)	1(50.0)	1(50.0	1(50.0)	1(50)	0	0	0	
	I	0	0	0	0	0	0	0	0	0	0	0	
	R	0	0	0	1(50)	1(50)	1(50)	1(50)	1(50)	2(100	2(100	2(100	
Serratia spp (n = 2)	S	2(100)	2(100)	2(100)	2(100)	0	1(50)	1(50)	2(100.0	0	0	1(50)	
	I	0	0	0	0	0	0	1(50)	0	0	0	0	
	R	0	0	0	0	2(100)	1(50)	0(0.0)	0	2(100)	(100)	1(50)	
K.ozaenae (n = 1)	S	1(100)	1(100)	1(100)	1(100)	0	0	0	1(100)	0	0	0	
	I	0	0	0	0	0	1(100	1(100	0	0	0	0	
	R	0	0	0	0	1(100)	0	0	0	1(100)	1(100)	1(100)	
Total (n = 25)	S	19(76)	19(76)	22(88)	15(60)	5(23.8)	7(28)	7(33.3)	11(52.4)	7(28)	2(8)	3(14.3)	
	I	0(0)	1(4)	1(4)	0(0)	0(0)	1(4)	2(9.5)	1(4.8)	1(4)	0(0)	0(0)	
	R	6(24)	5(20)	2(8)	10(40)	16(76.2)	17(68)	12(57.2)	9(42.8)	17(68)	23(92)	18(85.7)	

**Key**: −GNB: Gram−negative bacteria, MER: Meropenem, IPM: Imipenem, AMK: Amikacin, CPR: Ciprofloxacillin, CTR: Ceftriaxone, GM: Gentamycin, C: Chloramphenicol, TS: Trimethoprin−Sulfamethoxazole, CAZ: Ceftazidime, AMP: Ampicillin, AMC: Amoxicillin−clavulanic acid PIP: pipracillin, S: Sensitive, I: Intermediate, R: Resistance, Nr: Not recommended

### Gram-positive bacteria

The antimicrobial sensitivity pattern results showed that Gram-positive bacteria, *S.aureus* 11(91.7%) was resistant to penicillin, 5(41.7%) was resistant to gentamycin, and 7 (58.3%) was resistant to ciprofloxacin. From 12 *S.aureus*, 8 (66.7%) were methicillin resistance *Staphylococcus* (MRSA) by using cefoxitin disc whereas the remaining four (33.3%) were methicillin-sensitive *S. aureus* (MSSA).In other words, CONS were only one (25%) methicillin resistance, and three (75%) were methicillin-susceptible coagulase-negative *staphylococci*. *S.pyogenes* were (100%) susceptible to all antimicrobials used. *S.agalactia* was (100%) sensitive to ceftriaxone, trimethoprin-sulfamethoxazole and vancomacin, however (50%) resistance to ampicillin (Table 4).

**Table 4:** Antimicrobial susceptibility pattern of Gram-positive bacteria isolated from blood culture for patients suspected of septicemia at a hospital, in Northwest Ethiopia, 2021

Gram positive bacteria		Antimicrobial disks								
		PEN	GM	CPR	CXT	CFP	CTR	AMP	TS	VAN
*S.aureus* *(n = 12)*	S	1(8.3)	9(75)	10(83.3)	4(33.3)	Nr	Nr	Nr	Nr	Nr
	I	0	0	0	0	Nr	Nr	Nr	Nr	Nr
	R	11(91.7)	3(25)	2(17.3)	8(66.7)	Nr	Nr	Nr	Nr	Nr
CONS (n = 4)	S	3(75)	3(75)	3(75)	3(75)	Nr	Nr	Nr	Nr	Nr
	I	0	0	0	0	Nr	Nr	Nr	Nr	Nr
	R	1(25)	1(25)	1(25)	1(25)	Nr	Nr	Nr	Nr	Nr
*S.pyogenes* *(n = 2)*	S	2(100)	Nr	Nr	Nr	2(100)	2(100)	2(100)	Nr	2(100)
	I	0	Nr	Nr	Nr	0		0	Nr	0
	R	0	Nr	Nr	Nr	0	0 0	0	Nr	0
*S.agalactia* *(n = 2)*	S	Nr	Nr	Nr	Nr	Nr	2(100)	1(50)	2(100)	2(100)
	I	Nr	Nr	Nr	Nr	Nr	0	0	0	0
	R	Nr	Nr	Nr	Nr	Nr	0(0.0)	1(50)	0	0
Total (n = 20)	S	6(33.3)	12(75)	13(81.3)	7(43.7)	2(100)	4(100)	3 (75)	2(100)	4(100)
	I	0(0.0)	0	0	0	0	0	0	0	0
	R	12(66.7)	4(25)	3(18.7)	9(56.3)	0	0	1(25)	0	0

**Key: −***CONS: Coagulas−enegative Staphylococcus*, PEN: Penicillin, GM: Gentamicin, CPR: Ciprofloxacillin, CXT: Cefoxitin, CFP: Cefepime, CTR: Ceftriaxone, AMP: Ampicillin, TS: Trimethoprin−Sulfamethoxazole, VAN: Vancomacin, S: Sensitive, I: Intermediate, R: Resistance, Nr: *Not recommended*

### Multidrug resistance (MDR)

The prevalence of multidrug resistance in our study was 27/45 (60%). Whereas three (6.7%) blood bacterial isolates were sensitive to all antimicrobials tested, one isolated bacteria was resistant to all antibiotic groups tested. However, when drug resistance patterns were compared to species-specific observed, two (100%) of *k.rhinose*, two (100%) of *Serratia spp*, six (75%) of *K.pneumonia*e and three (75%) of *P.aeruginosa* were MDR pathogens in this study **(**Table 5**).**

**Table 5:** Multi-drug resistance patterns of bacteria isolated from septicemia suspected patients at a hospital, in Northwest Ethiopia, 2021

Bacteria isolate	Antimicrobial Resistance pattern N (%)								
	R0	R1	R2	R3	R4	R5	R6	R7	MDR
*S.aureus*	1(8.3)	3(25)	1(8.3)	2(16.7)	5(41.7)	0(0)	0(0)	0(0)	7(58.3)
CONS	0(0)	3(75)	0(0)	0(0)	1(25)	0(0)	0(0)	0(0)	1(25)
*S.pyogenes*	0(0)	2(100)	0(0)	0(0)	0(0)	0(0)	0(0)	0(0)	0(0)
*S.agalactia*	0(0)	0(0)	1(50)	1(50)	0(0)	0(0)	0(0)	0(0)	1(50)
*K.pneumonia*	1(12.5)	1(12.5)	0(0)	2(25)	1(12.5)	1(12.5)	2(25)	0(0)	6(75)
*E.coli*	1(16.7)	0(0)	1(16.7)	0(0)	1(16.7)	1(16.7)	1(16.7)	1(16.7)	4(66.7)
*P.aeruginosa*	0(0)	1(25)	1(25)	2(50)	0(0)	0(0)	1(25)	0(0)	3((75)
*E.aerogenes*	0(0)	0(0)	1(50)	0(0)	0(0)	1(50)	0(0)	0(0)	1(50)
*K.rhinose*	0(0)	0(0)	0(0)	1(50)	0(0)	0(0)	1(50)	0(0)	2(100)
*Serratia spp*	0(0)	0(0)	0(0)	1(50)	1(50)	0(0)	0(0)	0(0)	2(100)
*K.ozane*	0(0)	0(0)	1(100)	0(0)	0(0)	0(0)	0(0)	0(0)	0(0)
Total	3(6.7)	10(22.2)	6(13.3)	9(20)	9(20)	3(6.7)	5(11.1)	1(2.2)	27(60)

**Key:−**RO: Sensitive for all antimicrobial groups, R1: resistance for only one antimicrobial groups, R2: resistance for two antimicrobial groups, R3: resistance for three antimicrobial groups, R4: resistance for four antimicrobial groups, R5: resistance for five antimicrobial groups, R6: resistance for six antimicrobial, and R7: resistance for seven antimicrobial groups

## Discussions

The findings of this study revealed that the overall prevalence of bacteria isolated from septicemia suspected patients via the blood culture method was 21%, which was consistent with studies conducted in a variety of countries around the world, including Ethiopia, India, (16.08–22.3%) [20, 22, 32, 33], Jimma (18.2%) [1], Gondar (18.2%,19.4%)[6, 11], Mozambique (15.1%) [34], Iran (21.1%) [35], Ghanaian hospital (23.16%) [25], Nigeria (20.4%) [36]. On the other hand, lower than the study conducted in India (90%,30.6)[27, 37] Iran (38%) [38], Cameroon (28.3%) [24], Addis Ababa (27.9%,32.8%)[39, 40], Mekelle (28%) and Bahir Dar (32%, 39.2%) [41, 42]. However, higher among studies done in AddisAbaba (13%) [10], Jimma (8.8%) [43], Arba Minich (11.3%) [28], Nepal (10.6%) [14], Kuwait (2.3%) [19], New Zealand (9.5%) [44] India (4.4%) [45], Bangladesh (13.2%)[45], Ghana (11.2%) [46], and Southern Africa (5.5%) [47]. The possible reason for this variation might be explained by the fact that difference in the geographical location, the study population, method of study design, sample size, epidemiological variation, implementation of infection prevention and control, blood culture diagnostic system, study duration, and health care policy systems in these different countries.

In the present study, Gram-negative bacteria were more predominantly isolated than Gram-positive bacteria (55.6%) and (44.4%), respectively. Similarly, concordant findings were observed in India (51.2%) [37] Iran (55.4%)[35] Nepal (52.3%, 50.5%) [14, 48]. However, our finding was lower than other studies conducted, where Gram-positive bacteria was the predominant isolated bacteria than Gram-negative bacteria in India (60.9%61.4%) [43], Jimma (53.3%) [1], Addis Ababa (77.4%) [39], Gondar (66%) [11]. The varying percentages could be due to methodology difference and the patient safety practice, diagnostic method and sample size. In the current study the prevalence of *S. aureus* (26.7%), *K.pneumoniae (17.8%), E. coli (13.3%)*, CONS (8.9%), and *P.aeruginosa* (8.9%) were the main isolate, this was in line with Indonesia *K.pneumoniae* (17%), *P.aeruginosa* (12%) [49]. Greater or a lesser amount of similar results have been seen in studies conducted in septicemia suspected patients from different regions in Ethiopia, Addis Ababa (50%, 26.21% 14.02%) [39], Mekelle (37.5%,11.1%) [12], and Southern Ethiopia (32.2%,14.3%) [28]. Due to these bacteria are normal microbiota of the skin, and the majority of the time found in the hospital areas as a result cause infection in immune susceptible patients.

In the present study, different associated factors were evaluated for various associations with septicemia. Nevertheless, not all of the socio-demographic variables in the current study were statistically associated with septicemia. However, this study indicated that males were more infected than females (23.8% vs. 19.2%), respectively, but there was no a statistically significant difference in gender variation (p = 0.632). This slight variation has been reported in different studies [11, 12, 50]. Patients in under-five age group were more infected with septicemia as compared to the adult age group. The possible reason for this is the children have more susceptible to infection due to less immune system and colonized by normal microbiota. Nevertheless, in this study the category of age groups were not statistically significant (p = 0.186). This finding disagrees with the research done in southern Ethiopia [28], Rwanda [51] Nepal [14], and the USA [52]. The possible reason for this difference might be due to the none representative of samples in each ward from this study. Previous hospitalization in this study was 2.9 times more likely associated with septicemia in the current study. This research finding is consistent with the study conducted in Ethiopia AOR = 5.54 and AOR = 3.2 [8, 28]. This association might be due to health care associated infections, high number of patients admitted to hospitals for prolonged periods, delayed request for blood culture, and a weak patient safety, and management system in the hospital. As a result, fast intervention had a must to minimize the infection.

The overall resistance of Gram-positive and Gram-negative bacteria in this study were 25-66.7% and 8-92% respectively, which was similar to the result reported in Mekelle [12], Jimma [1]. This condition gives severe anxiety to the population. This high rate of resistance might indicate misuse and inappropriate usage of the antibacterial drugs. However, the resistance is different from the study conducted in Gondar where the rate of resistance for Gram-positive bacteria ranged from (23.5- 58.8%) and Gram-negative bacteria (20% 100%) [11]. The observed variation may be due to the arbitrary use of antimicrobials in the study area, self-prescribing antimicrobials in private drug stores and empirical treatment of hospital acquired infections without real etiological agent identification.

*S.aureus* was (91.7%) resistant to penicillin, but susceptible to (75%) gentamicin, and (83.3%) ciprofloxacillin. This was similar with reports conducted in different countries 40-97% [10, 20, 33]. This is due to the unrestrained use of antimicrobials without sensitivity testing in the study area. (66.7%) of MRSA was detected. In order of, the same report was done in Indian and Ethiopian researches [6, 20, 45]. The incident of MRSA is more common because of the haphazard use of higher antimicrobials empirically and genetic proficiency to obtain antibiotic resistance. *S. pyogenes* were 100% sensitive to cefepime, ampicilin, and vancomycin from our study. This was in line with the study reports, which had in 100%, 87% [13, 33]. Vancomycin was 100% sensitive to *S. agalactia.* This finding was similar to the reports done in Mekelle [12]. The possible reason for the absence of vancomycin resistant bacteria in this study is that the use of vancomycin by clinicians is controlled in the management of patients in the study setting, and in the nation as an entire.

*K.pneumoniae, E.coli, P.aeruginosa, K.rhinose*, and *Serratia spp* in this study were (50-100%) sensitive to meropenem, imipenem, and amikacin. This finding was similar to the reports [20, 28, 37, 45]. *K.pneumoniae* and *E.aerogenes* were 100% resistant to ampicillin, amoxicillin -clavulanic acid. In addition, *E.coli* (83.3%) for gentamicin and *P.aeruginosa* (75%) for gentamicin and ampicillin resistance were observed in this study. These were consistent to other studies conducted in Ghana 74% [46], Ethiopia 86% and 94% [8, 41], India 93%, 87% [45, 53]. The finding of this study indicates most of the gram-negative bacteria were 50–100% resistance to ciprofloxacillin, gentamicin, ceftriaxone, chloramphenicol, trimethoprin-sulfamethoxazole, and ceftazidime. This was concordant with the research done in Cameroon 45-100% [24], and Ethiopia [8, 41, 43]. The limitation of this study was done in a single hospital, small sample size, short duration and only a phenotypic test was done, which may under estimate the prevalence of bacteria for the different geographical areas. This study has not detected fungemia and anaerobic bacteria due to scarcity of the media.

## Conclusions

In this study, the overall prevalence of blood culture positivity among septicemia suspected patients was moderately high. Gram-negative bacteria were the predominant etiological agents for septicemia in the study populations. *S.aureus, K.pneumoniae, E.coli*, CONS, and *P. aeruginosa* were the predominant causative agent for septicemia. Conversely, *S. aureus* was the highest prevalence in the bacterial isolates. Previous hospital admission, fever, and length of hospital stay were significantly associated with septicemia in our study. Gram-negative bacteria were highly resistant to ampicillin, amoxicillin-clavulanic acid, ceftriaxone, chloramphenicol, and trimethoprim-sulfamethoxazole, but susceptible to meropenem, imipenem and amikacin. *S. aureus* was highly resistance to penicillin. While, susceptible to gentamicin and ciprofloxacillin. High multi-drug resistance rates were observed in most bacterial isolates. The study calls researchers to conduct a further study with a large sample size, with better study design and budget. Clinicians are better to establish hospital antibiotic stewardship to minimize antimicrobial resistance among septicemia suspected patients. It is also essential to conduct constant antimicrobial sensitivity surveillance on blood culture isolates and ensuring more rational antimicrobial use and a combination of antimicrobial therapy may help to verify the appearance of resistance.

## Data Availability

All relevant data were present within the manuscript. The datasets used during the current study are available from the corresponding author on reasonable request.

## Abbreviations

AMR: Antimicrobial Resistance
ATCC: American Type Culture Collection
SBAP: Sheep Blood agar plate
BSIs: Blood Stream Infections
CAP: Chocolate agar plate
CLSI: Clinical Laboratory Standards Institute
CONS: Coagulase-negative Staphylococcus
DMCSH: Debre Markos Comprehensive Specialized Hospital
ICU: Intensive Care Unit
LMICS: Low and Middle Income Countries
MAC: MacConkey Agar
MDR: Multidrug Resistance
MHA: Mueller Hinton Agar
MSA: Mannitol Salt Agar
QC: Quality control
SOPS: Standard Operating Procedures
SPSS: Statistical package of social science
TSB: Trypton soy broth
WHO: World health organization
